# Orthostatic Hypotension in Asymptomatic Patients with Chronic Kidney Disease

**DOI:** 10.3390/medicina55040113

**Published:** 2019-04-20

**Authors:** Beata Januszko-Giergielewicz, Leszek Gromadziński, Maria Dudziak, Alicja Dębska-Ślizień

**Affiliations:** 1 Family Medicine Unit, School of Medicine, Collegium Medicum, University of Warmia and Mazury in Olsztyn, ul. Warszawska 30, 10-082 Olsztyn, Poland; 2 II Clinical Department of Cardiology and Internal Medicine, Faculty of Medicine, Collegium Medicum, University of Warmia and Mazury in Olsztyn, ul. Warszawska 30, 10-082 Olsztyn, Poland; lgol@op.pl; 3 Cardiac Diagnostic Unit, II Department of Cardiology, Medical University of Gdańsk, ul. Mariana Smoluchowskiego 17, 80-214 Gdańsk, Poland; mdudziak@gumed.edu.pl; 4 Clinical Department of Nephrology, Transplantology and Internal Medicine, Medical University of Gdańsk, ul. Dębinki 7, 80-211 Gdańsk, Poland; adeb@gumed.edu.pl

**Keywords:** orthostatic hypotension, chronic kidney disease

## Abstract

*Background and objective*: Orthostatic hypotension (OH) is a decrease in systolic blood pressure (BP) of 20 mm Hg and in diastolic BP of 10 mm Hg when changing the position from lying to standing. Arterial hypertension (AH), comorbidities and polypharmacy contribute to its development. The aim was to assess the presence of OH and its predictors in asymptomatic chronic kidney disease (CKD) patients. *Material and methods*: 45 CKD patients with estimated glomerular filtration rate (eGFR) ≤ 60 mL/min/1.73 m^2^ (CKD+) were examined for signs of OH and its predictors. The results were compared with the control group of 22 patients with eGFR > 60 mL/min/1.73 m^2^ (CKD–). Asymptomatic patients without ischemic heart disease and previous stroke were qualified. Total blood count, serum creatinine, eGFR, urea, phosphates, calcium, albumins, parathyroid hormone, uric acid, C reactive protein, N-terminal pro b-type natriuretic peptide, lipid profile, and urine protein to creatinine ratio were assessed. Simultaneously, patients underwent echocardiography. To detect OH, a modified Schellong test was performed. *Results*: OH was diagnosed in 17 out of 45 CKD+ patients (average age 69.12 ± 13.2) and in 8 out of 22 CKD– patients (average age 60.50 ± 14.99). The CKD+ group demonstrated significant differences on average values of systolic and diastolic BP between OH+ and OH– patients, lower when standing. In the eGFR range of 30–60 mL/min/1.73 m^2^ correlation was revealed between OH and β-blockers (*p* = 0.04), in the entire CKD+ group between β-blockers combined with diuretics (*p* = 0.007) and ACE-I (*p* = 0.033). Logistic regression test revealed that chronic heart failure (CHF, OR = 15.31), treatment with β-blockers (OR = 13.86) were significant factors influencing the presence of OH. *Conclusions*: Predictors of OH in CKD may include: CHF, treatment with β-blockers, combined with ACE-I and diuretics.

## 1. Introduction

Orthostatic hypotension (OH) is a pathological condition defined as a decrease in systolic blood pressure (BP) of 20 mm Hg (30 mm Hg according to some authors) or more and a decrease in diastolic BP of 10 mm Hg or more when the body position is changed from lying to standing [1]. This is caused by an impaired adaptation of the circulatory system to the change in body position, and results from the autonomic nervous system (ANS) dysfunction [2]. The presence of OH is the main symptom of autonomic neuropathy (AN) and may be its indirect indicator [2]. Physiologically, assuming a standing position causes significant, gravitation-dependent changes in the circulatory system. Several adaptive mechanisms ensure that BP is retained at an adequate level. This occurs via the reflex arc by stimulating ANS baroreceptors, resulting in the vessels constriction in the venous bed and blood redistribution to increase its inflow to the heart [1,2,3]. Another adaptive mechanism is increased muscle tension in the lower extremities in the standing position and decreased pressure in the chest [3]. Additionally, the activity of the adrenergic system is increased, the renin angiotensin aldosterone system (RAA) is activated, and vasopressin secretion is enhanced. These neurohormonal phenomena lead to increased resistance of blood vessels and increased heart rate as well as kidney retention of sodium and water. Due to all these mechanisms average arterial pressure reaches values close to normal in the standing position [3]. OH results from impaired adaptation of the circulatory system to the standing position, and pathological changes may affect all the organs and systems described above. Blood in volumes of 500–700 mL, and even approaching 1.5 L in the vessels below the diaphragm, pools in the lower sections of the venous system, thus decreasing inflow to the heart and cardiac output, and causing a drop in arterial pressure in the standing position [3,4]. OH is mainly caused by prolonged immobility, advanced age, pregnancy, and gastrectomy [4]. Its clinical manifestation has a wide spectrum, ranging from very mild symptoms, i.e., weakness, dizziness and fainting, to serious conditions accompanied by loss of consciousness. Within the spectrum of civilization diseases, OH coexists with arterial hypertension (AH), metabolic disorders like diabetes, chronic kidney disease (CKD), dialysis therapy, or toxemia with secondary damage to the ANS (long-term alcohol-dependence, long-term drug use, drug induced damage), and electrolyte imbalance [5,6,7]. A model disease that illustrates this problem is diabetes, and the coexistence of OH and AN in diabetic patients has been quite thoroughly researched [5,8,9]. Diabetic autonomic neuropathy is a serious and common complication of diabetes, and cardiovascular autonomic neuropathy (CAN) is its most studied and clinically important form, since it is associated with an increased risk of mortality due to cardiovascular (CV) causes. The determination of the presence of CAN is usually based on a battery of the ANS function tests, and consequently this condition frequently remains undiagnosed [6]. At present, a battery of three tests is recommended: heart rate variability (HRV), the Valsalva maneuver, and postural blood pressure testing [10].

It has been commonly known for many years that peripheral neuropathy is one of the major complications in CKD patients, and is found in nearly half of patients who receive dialysis [10,11]. It is probably responsible for the absence of angina in CKD, and an asymptomatic form of ischemic heart disease (IHD)—silent myocardial ischemia (SMI)—dominates in this population of patients. The authors of this paper have been interested for several years in finding practical tools for AN assessment in asymptomatic patients with CKD [12]. The number of available reports concerning the presence of OH in dialysis patients, especially patients receiving hemodialysis (HD), is vast [13,14]; however, studies on the development of OH and AN in patients in the early stages of CKD are lacking.

The aim was to assess the presence of OH and its predictors in asymptomatic CKD patients. 

## 2. Materials and Methods 

In total, 45 patients from the Nephrology Outpatient Clinic were examined (average age 66.98 ± 12.52 years, 23 females and 22 males), with CKD and estimated glomerular filtration rate (eGFR) ≤ 60 mL/min/1.73 m^2^, indicating CKD stage 3–5 according to the NKF KDOQI classification as of 2002 (National Kidney Foundation—Kidney Disease Outcomes Quality Initiative) [12], further termed renal failure group—CKD+. Stage 3 CKD (eGFR 59–30 mL/min/1.73 m^2^) was detected in 28 patients, stage 4 (eGFR 29–15 mL/min/1.73 m^2^) in 16 patients, and stage 5 CKD (eGFR < 15 mL/min/1.73 m^2^) in 1 patient. The patients were examined for signs of OH. Patients with OH were termed OH+, without OH were termed OH–.

Examination results and the presence of OH were compared with a group of 22 patients with eGFR > 60 mL/min/1.73 m^2^ (average age 61.14 ± 14.35 years, 14 females and 8 males), further termed CKD–. Only patients with no clinical manifestations and no history (patient’s complaints and overview of medical history) of IHD (angina, CV incidents, and hospitalizations), no history of previous stroke and transient ischemic attack were qualified for the study. Additional exclusion criteria included: clinically diagnosed infections, hemorrhages, newly diagnosed and/or treated cancer, and psychiatric disorders, precluding the patient’s conscious consent and/or cooperation. 

All patients were examined to assess total blood count, serum levels (using BA 400 device) of creatinine (Jaffe’s reaction), eGFR calculated with the modified MDRD formula, levels of urea, phosphates, calcium, albumins, parathyroid hormone (PTH), uric acid, triglycerides, HDL-cholesterol, LDL-cholesterol, C reactive protein (CRP), and urine protein to creatinine ratio. Moreover, N-terminal pro b-type natriuretic peptide (NT-proBNP) level was assayed using the Startus CS Acute Care immunological test (Simens, Munich, Germany).

Apart from laboratory tests, patients simultaneously underwent echocardiography (ECHO). ECHO was conducted using a GE 6S (GE, Fairfield, CT, USA) device equipped with a 2.5–3.5 MHz transducer. The following parameters were assessed: left ventricular (LV) end-diastolic diameter (LVEDD, cm), interventricular septal diastolic diameter (IVSd, cm), posterior wall thickness in diastole (PWTd, cm), and left atrial dimension (LAD). Left ventricular ejection fraction (LVEF) was assessed in a four-chamber view using the simplified Simpson method. Left ventricular mass (LVM) was calculated on the basis of the formula recommended by the American Society of Echocardiography (ASE) as modified by Devereux [15]. The obtained LVM results were indexed by the body surface area of the patient and presented as the left ventricular mass index (LVMI). Peak early diastolic transmitral flow velocity (E), peak late diastolic transmitral flow velocity (A) and E/A ratio were assessed using pulse wave Doppler in a four-chamber view as developed by Devereux [16,17].

The level of comorbidities was assessed for all patients using the modified Davies comorbidity index [18], accounting for co-existent diseases, i.e., cancers, peripheral arteries disease, LV failure (measured in this study as a value of LVEF ≤ 60%), diabetes, systemic disease, and lung diseases. 

To detect OH, a modified Schellong test (active standing test) was performed [1]. It was conducted in two stages:

- Stage I—measure of BP in a lying position, after a minimum of 3–5 min rest,
- Stage II—measure of BP in a standing position after 2–3 min,
- Stage III, i.e., a repeated measure after assuming a lying position, was not performed.

Statistical analysis was performed using Statistica 12.0 PL software (StatSoft, Tulsa, OK, USA). Interval, ordinal, and nominal data were analyzed. The Shapiro-Wilk *W* test was used to test the distribution of interval data for normality. To compare normally distributed interval data characterized by homogeneity of variance (verified by Levene’s test) Student’s *t*-test was used for independent variables. One-way analysis of variance (one-way ANOVA) (Fisher’s test) was used to compare more than two mean values. When this test yielded statistically significant results, post hoc comparisons were performed using Tukey’s HSD (honestly significant difference) test.

Interval data of non-normal distribution were compared between two groups using a non-parametric Mann-Whitney *U* test. In the case of non-normal distribution (right-skewed distribution), for some groups of data the distribution was successfully normalized through the calculation of natural logarithms. 

Nominal data were compared with the chi-squared test or, when it was justified, with Cramer’s *V* or Yates’s chi-squared tests. For the analysis of an arrangement of three variables the chi-squared Mantel-Heanszel test was applied.

Logistic regression was used for identifying risk factors for the presence of OH, with quasi-Newton method used for estimation, and quasi-Newton method and Rosenbrock’s function when estimation was uncertain. Nominal data were coded as follows: 0—absence of the factor, 1—presence of the factor. After performing a univariate analysis, variables that obtained a statistical significance of less than 0.05 in a univariate analysis were included in a multivariate analysis in order to identify independent risk factors. Backward stepwise regression was applied to analyze data. To verify all statistical hypotheses, we assumed a statistical level of significance of *p* < 0.05. 

All patients consented in writing for inclusion in the research. The study protocol was approved by the Bioethics Committee (no 49/2011/IV).

## 3. Results

During the first stage of the study, a descriptive analysis of the examined patients was performed. Table 1 presents general clinical characteristics of the study group. In the CKD+ group, OH was detected in 17 out of 45 (37.8%) patients (average age 69.12 ± 13.2 years), and in the CKD– group in 8 out of 22 (36.3%) patients (average age 60.50 ± 14.99 years). Post hoc tests revealed significant differences in the mean values of systolic and diastolic BP between OH+ and OH– groups, lower in the standing position. 

Table 2 includes the biochemical characteristics of both groups. The CKD+ group, as compared to the CKD– group, presented statistically higher levels of CRP, PTH, NT-proBNP, uric acid, urine protein to creatinine ratio, and lower hemoglobin (Hb) levels. Statistically interesting data were obtained in the post hoc test for Hb levels in the subgroup with eGFR ≤ 29 mL/min/1.73 m^2^ between the OH+ and OH– patients (*p* = 0.032). Parameters obtained in ECHO for both groups are presented in Table 3. The CKD+ group had statistically significantly lower values of the E/A ratio and higher values of IVSd. Post hoc tests did not reveal statistically significant differences of mean values between the groups.

Table 4 includes a comparison of pharmacological therapies in the groups. The number of patients is 64 because 3 patients did not receive any of the drugs from the enumerated groups. A statistically significant relationship was revealed between the presence of OH and treatment with β-blockers (*p* = 0.04) for the eGFR range of 30–60 mL/min/1.73 m^2^. 

We also investigated the possibility of determining a possible aggregation of clinical risk factors for OH in the CKD+ group. Interesting statistically significant correlations were revealed when associations between variables were tested (using Yates’s chi-squared test or Cramer’s *V* test). These relationships are presented in Table 5, Table 6 and Table 7. This method demonstrated the impact of treatment with β-blockers, especially combined with diuretics (Table 5), on the presence of OH in the CKD+ group (*p* = 0.007).

Treatment with β-blockers was also found to be a significant predictor of OH, when this phenomenon was analyzed in aggregation in both groups (Table 6). This impact was particularly evident in combination with angiotensin-converting enzyme inhibitors (ACE-I) and decrease in eGFR ≤ 60 mL/min/1.73 m^2^ (*p* = 0.033) in the presence of OH, similarly to a combination of treatment with β-blockers with congestive heart failure (CHF) and eGFR ≤ 60 mL/min/1.73 m^2^ (*p* = 0.005), as well as other correlations. With respect to sex differences, some significant association rules were found for males. These are also collected in Table 7. A statistically significant correlation between OH and simultaneous treatment with ACE-I and eGFR ≤ 60 mL/min/1.73 m^2^ was revealed for male patients (*p* = 0.02), as well as between these variables and comorbidity >1 (0.013). No statistically significant correlations were revealed for females (perhaps due to a small sample size). 

Logistic regression was used for identifying risk factors for OH in the CKD+ group. A model including the following parameters was obtained:

Dependent variable:

- OH presence (0—absence, 1—presence).

Independent variables:

- CHF presence (0—absence, 1—presence),
- β-blocker treatment (0—absence, 1—presence),
- systolic BP in the standing position (quantitative variable),

Table 8 presents the results of multivariate logistic regression analysis in the CKD+ group. 

The most significant factor that influenced the presence of OH was CHF (odds ratio (OR) for a unit change = 15.31). Treatment with β-blockers was found to be a weaker factor (OR for a unit change = 13.86). The weakest impact for the development of OH was revealed for the systolic BP values in the standing position (OR for a unit change = 0.91).

## 4. Discussion

In the CKD+ group, OH was detected in 17 out of 45 (37.8%) patients, and in the CKD– group in 8 out of 22 (36.3%) patients. Although percentage differences between the study group and the control group were not significant, OH was found in every third patient in the CKD+ group, which indicates the scale of the problem. Post hoc tests revealed significant differences in the mean values of systolic and diastolic BP in the CKD+ group between OH+ and OH– patients, lower in the standing position. A strong tendency towards drops in BP values after assuming a standing position was revealed, though not all cases met the OH criteria. This may evidence worse conditions for the stabilization of the circulatory system upon assuming a standing position and a high probability of the development of CAN in patients with CKD.

According to available reports, OH is found in approx. 25–30% of individuals older than 65 years [4], and in randomly selected cohorts of asymptomatic patients with diabetes it was detected in approx. 20% of patients [5]. As already mentioned in the introductory section, OH is a well-documented, primary symptom of AN [5], which is best described in diabetic patients as diabetic autonomic neuropathy. CAN, the most important form of AN with respect to death risk, still remains underappreciated [9,18,19]. Two large prospective studies analyzed the relationship between CAN and future mortality or morbidity, i.e., myocarditis, CHF, ventricular tachycardia or ventricular fibrillation, IHD or necessity for revascularization. The relative risk amounted to 2.2, 3.4, 18 and 25, respectively. In a study published in 2006, it was shown that in a long-term observational follow-up, CAN is an independent predictor for cardiovascular morbidity and mortality in type 1 diabetic patients with diabetic neuropathy [20]. A meta-analysis of 15 studies, in which 2900 diabetic patients were observed, reveals that CAN increases relative death risk by 2.14-fold [20]. 

In our study, every third examined patient with OH and CKD had diabetes, which is in line with the data from the literature [9,19,20]. Numerous clinical studies indicate that CAN coexists with SMI [12,19]. We included only asymptomatic patients in our study, searching for OH in this specific patient group.

CAN results in the decreased conductivity of central sympathetic signals and a significant coronary blood flow reduction [21]. Additionally, this process is exacerbated by oxidative stress and production of free radicals. Free radicals cause endothelial damage and reduce the bioavailability of nitric oxide. Immune factors also play a significant role in CAN [21]. Many studies indicate HRV as a very useful test to assess the ANS dysfunction, especially in early, subclinical phases [10]. Meta-analyses of the published data demonstrate that a reduced CV function measured by HRV is closely associated with an increased risk of silent myocardial infarction and mortality [22]. 

OH is also a well-known symptom in the everyday practice of nephrologists. However, AN has not been sufficiently researched, although it is a common complication of an end-stage CKD. Only a few studies devoted to this problem are available, mainly case reports describing patients with diabetes and renal complications. AN in CKD, as in other metabolic disorders, begins silently and then progresses insidiously for months and years. It most frequently develops at GFR < 12 mL/min/1.73 m^2^. It is estimated, however, that this complication is present in 60–100% of patients receiving dialysis [7]. In our study, we demonstrated that OH is also found in the earlier stages of CKD, which can be explained by the presence of other common risk factors for the development of OH, i.e., ageing of the population of CKD patients, increase of comorbidities and of several administered drugs. A larger group with end-stage CKD would definitely further increase these proportions (in our study only 1 patient had eGFR < 15 mL/min/1.73 m^2^).

The development of uremic neuropathy had previously been related to the retention of neurotoxic molecules in the middle molecular range, although this hypothesis lacked solid proof. Recent studies have demonstrated that the nerves of uremic patients exist in a chronically depolarized state, and are additionally characterized by a delayed normalization of resting membrane potential after HD [7]. The degree of depolarization correlates with serum potassium concentration, suggesting that maintenance of serum potassium within normal limits is likely to reduce the severity of AN in HD patients [7,13]. Dialysis-induced hypotension (DIH), a clinically important form of AN in CKD, should also be mentioned. This condition often manifests itself as OH. DIH is present in 15–30%, and even 50%, of HD sessions [23], and not only significantly reduces quality of life, but is also an independent predictor of mortality in the population of dialysis patients [24]. It is commonly known that, in patients with CKD, AN is mainly related to enhanced sympathetic activity [12]. There is ample evidence for the increased activity of the sympathetic system in CKD patients. Based on microneurography of the peroneal nerve, Convers et al. demonstrated increased neurotransmission in this patient group [25]. It should be remembered that in patients in advanced stages of CKD with symptoms of dehydration and/or excessive urinary sodium excretion, OH may be more acutely manifested due to progressive long-term dehydration. 

A long-term increase in sympathetic activity leads to the development of diseases significant to survival, i.e., AH, IHD and LV hypertrophy [25,26]. As previously mentioned, patients with CKD and/or diabetes frequently manifest resting tachycardia, most often due to vagal neuropathy that induces sympathetic nerves activity. Tachycardia is a direct indication of the presence of hyperkinetic circulation, additionally induced by an arteriovenous fistula formed to administer HD and anemia. Hyperkinetic circulation further changes the sympathetic-parasympathetic balance, privileging the former [27], and thus closing the vicious circle of an enhanced sympathetic function in patients with CKD. Tachycardia is easy to assess and indirectly provides information as to the level of sympathetic system activation. At present it is recognized as an independent predictor of arrhythmia and sudden cardiac death not only in this group of patients, but also in the general population [28]. Many reports indicate that abnormalities in HRV in CKD patients are caused by AN. There are also data demonstrating that lowered values of HRV in CKD patients are related to the risk of sudden cardiac death in this patient group [29,30]. Lawrence et al. described three cases of sudden incidents of bradycardia and hypotension in non-dialysis patients with CKD and diabetes [31]. Their resting heart rate was constantly elevated to a range of 101–110 bpm, and did not change during deep breathing or the Valsalva maneuver. Changes in BP during exercise were not noted, and BP significantly dropped in the standing position (we obtained similar results in our study). However, intraoperative hypotension and bradycardia were observed; they occurred suddenly with no apparent cause. Bradycardia persisted despite a large dose of atropine administered intravenously, suggesting vague nerve damage at the heart level. The results of postoperative tests of CV reflexes indicated serious impairments of the sympathetic and parasympathetic systems. 

In our study, in the CKD+ group, as compared to the CKD– group, we detected lower values of eGFR (36 ± 21.44 mL/min/1.73 m^2^) and Hb, and higher levels of CRP, PTH, NT-proBNP, uric acid, and urea protein to creatinine ratio. As is evident based on the collected data, the presence of OH correlates in various statistical analyses with parameters that are documented risk factors for CV complications in CKD, i.e., anemia, infections, lipid metabolism disorders, and elevated levels of NT-proBNP [12]. This is a characteristic combination of factors which intensify their respective activities and accumulate as CKD progresses, which may stimulate the presence of OH.

In our study, the CKD+ group demonstrated lower values of the E/A ratio and higher values of IVSd. This indicates that cardiac pathology, secondary to CKD and leading to CHF, may also intensify the symptoms of AN, including OH [26]. In our study, CHF was a significant factor that contributed to the presence of OH as revealed in logistic regression analysis (OR = 15.31).

Available reports emphasize the relationship between age and the presence of OH. In elderly patients, this condition results from lowered cardiac output, reduced function of baroreceptors, weaker self-regulatory mechanisms, decreased volumes of water in the organism and atherosclerotic lesions, especially in cerebral arteries [8,31]. In patients older than 60 years, a drop of systolic BP by approx. 7 mm Hg is a physiological phenomenon, but may be dangerous. In our study, the average age of patients with OH in the CKD+ group was higher by about 10 years as compared to the CKD– group (69.12 ± 13.20 vs. 60.50 ± 14.99 years). In the Gerodiab study, a 5-year, multicenter French prospective study, 987 patients with type 2 diabetes and an average age of 77 ± 5 years were examined [8]. The values of BP in the lying position and 1, 3, 5 min after assuming the standing position were measured; 301 patients (30.5%) manifested OH, average systolic and diastolic BP and pulse values at rest were higher in the group with OH as compared to the group without OH. Patients with OH demonstrated the higher likelihood of peripheral artery disease and amputations (33% vs. 24%, *p* < 0.05; 3.3% vs. 1.5%, *p* = 0.056). Multivariate analysis showed the relationship between OH and serious AH (*p* < 0.01), increasing waist to hip ratio (*p* < 0.05) and amputations (*p* < 0.05) [8]. AH is the main cause leading to OH [2,5,8]. 

The role of the increase in the variety of comorbidities with age should be highlighted, as well as the overlapping of diseases that potentially have neurovegetative destructive effects, i.e., diabetes, CKD, atherosclerosis, gout, infections, and malnutrition [32,33,34,35]. This is in particular observed in CKD in which comorbidities are complex and result directly from the primary disease. 

In our study, in the examined male patients we also demonstrated a significant statistical relationship between the presence of OH and comorbidity > 1, with a simultaneous treatment with ACE-I and eGFR ≤ 60 mL/min/1.73 m^2^ (*p* = 0.013). In our opinion, this confirms an impact of comorbidities on OH in CKD patients.

With regard to pharmacological factors, treatment with β-blockers, particularly when administered in patients with eGFR ranging from 30–60 mL/min/1.73 m^2^, turned out to be in our study a predictor of OH in CKD patients. Logistic regression test performed for the CKD+ group also revealed that treatment with β-blockers was a significant factor influencing the presence of OH (OR for a unit change = 13.86). Blood levels of parasympathetic neuromediators are elevated as CKD progresses. This is due to the decreased activity of enzymes from the monoamine oxidase and catechol-O-methyltransferase groups, which break down catecholamine. This results in an adaptive mechanism relying on the reduction of adrenergic receptor concentration on effector cells, which significantly increases the risk of sudden CV complications [25,36]. During emotional stress, vascular stress (e.g., fluctuations in arterial blood pressure and/or body fluid status), metabolic stress (e.g., hypoglycemia, underdialysis) natural balancing mechanisms fail because they are impaired due to a reduced receptor response [37]. Treatment with β-blockers further impairs the described balancing mechanisms, and the impact of this drug group on the presence of OH in CKD demonstrated in our study may indirectly suggest AN in CKD. We observed the impact of β-blockers on the development of OH particularly in combination with treatment with ACE-I and diuretic drugs. This therapeutic scheme common in the treatment of CKD appears to be an evident pathophysiological mechanism leading to OH since diuretics and dehydration activate the RAA system, thus augmenting the effects of ACE-I and β-blockers. This is confirmed by findings from other studies [4,5,6]. It should be stressed that polypharmacy and drug interactions are the most frequent risk factors for the development of OH [36]. It should be stressed that the impact of cumulative comorbidities and the number of administered medication (polypharmacy) is aggravated as CKD advances. 

In the large SPRINT study [38], with 8662 participants (mean age 68 years), Townsend et al. observed a decrease >20 mm Hg in systolic blood pressure upon standing in 5% of the study population. Female sex, taller height, more advanced kidney disease, current smoking, and several drug classes were associated with larger declines in blood pressure upon standing.

As we have already stressed, a significant limitation of the presented study that needs to be taken into account when analyzing the obtained results is a small number of the examined patients. This undoubtedly impacts on the conclusions and their univocality. The size of study population was directly related to the fact that the study was performed in the conditions of everyday clinical practice of the Nephrology Out-patient Clinic and inclusion criteria that excluded patients with IHD symptoms, history of stroke and other CV incidents, all very common in patients with CKD. Another problem is related to a statistical balance between the study group and the control group due to, consistent with literature reports, a different percentage of most frequent comorbidities, i.e., diabetes and AH in the group with eGFR ≤ 60 mL/min/1.73 m^2^, as compared to the group with eGFR > 60 mL/min/1.73 m^2^.

## 5. Conclusions

OH occurs in asymptomatic CKD patients, also in its early stages, especially when accompanied by advanced comorbidities and polypharmacy. Predictors of OH in CKD may include CHF, treatment with β-blockers, especially when combined with ACE-I and/or diuretic drugs.

The strength of this study lies in demonstrating the presence of OH and its predictors as potential symptoms of AN in asymptomatic patients with CKD since reports in scientific literature concerning this issue are scarce. However, we did not demonstrate a significantly higher incidence of OH in CKD patients as compared to the control group, which is most likely associated with a small study group size, further limited by inclusion criteria, i.e., no history of IHD and other CV incidents, because we aimed at analyzing OH in patients without IHD symptoms and CV complications.

CKD itself, especially as it becomes more advanced, and metabolic disorders associated with it, as well as polypharmacy, proved to be a strong predictor for OH. In our view, this issue is extremely important and requires conducting further studies on a larger population of patients. 

## Figures and Tables

**Table 1:** Clinical characteristics of both groups.

Parameter	Total *n* = 67	CKD+ Group *n* = 45		CKD– Group *n* = 22		*p*
		OH+ *n* = 17	OH– *n* = 28	OH+ *n* = 8	OH– *n* = 14	
Age, years ^a,b^	65.06 ± 13.33	69.12 ± 13.12	65.68 ± 12.19	60.50 ± 14.99	61.50 ± 14.54	0.537 ^f^
Systolic BP—lying position, mm Hg ^a,b^	141.90 ± 21.89	143.94 ± 23.59	142.11 ± 16.56	152.50 ± 38.82	132.93 ± 14.34	0.134 ^g^
Systolic BP—standing position, mm Hg ^a,b^	138.88 ± 22.68	121.29 ± 16.92	148.18 ± 21.79	136.25 ± 23.26	143.14 ± 19.20	0.073 ^f^
Diastolic BP—lying position, mm Hg ^c,d^	80 (80,90)	80 (73,90)	80 (78,90)	90 (80,95)	80 (80,85)	0.706 ^g^
Diastolic BP—standing position, mm Hg ^c,d^	80 (73,90)	70 (60,80)	88.5 (80,100)	80 (60,90)	85 (80,90)	0.413 ^f^
Sex F, *n* (%) ^e^	37 (55.22)	9 (52.94)	14 (50.00)	8 (100.00)	6 (42.86)	0.033 ^h^
AH, *n* (%) ^e^	48 (71.64)	16 (94.12)	21 (75.00)	5 (62.50)	6 (42.86)	0.018 ^h^
Diabetes, *n* (%) ^e^	16 (23.88)	6 (35.29)	8 (28.57)	2 (25.00)	0 (0)	0.072 ^h^

Comments: CKD+—patients with estimated glomerular filtration rate (eGFR) ≤ 60 mL/min/1.73 m^2^; CKD–—patients with eGFR >60 mL/min/1.73 m^2^; OH+—patients with orthostatic hypotension; OH–—patients without orthostatic hypotension; BP—arterial blood pressure values; AH—arterial hypertension; ^a^ Continuous variables of normal distribution; ^b^ numbers are given as mean ± SD; ^c^ continuous variables of non-normal distribution; ^d^ numbers are given as median (lower quartile, upper quartile); ^e^ categorical variables; ^f^ F-test; ^g^ log transformation, F-test; ^h^ chi-squared MH (Mantel-Haenszel) test.

**Table 2:** Biochemical characteristics of both groups.

Parameter	Total *n* = 67	CKD+ Group *n* = 45	CKD– Group *n* = 22	*p*
Creatinine, mg/dL ^a,b^	1.41 (0.92, 1.94)	1.66 (1.41, 2.32)	0.81 (0.77, 0.92)	<0.001 ^e^
eGFR, mL/min/1.73 m^2 a,b^	44 (29, 71)	36 (21, 44)	78 (71, 83)	<0.001 ^e^
Urea, mg/dL ^a,b^	51 (34, 74)	61 (50, 85)	30,5 (25, 34)	<0.001 ^e^
Triglycerides, mg/dL ^a,b^	133 (100, 188)	134 (104, 174)	135.5 (89, 212)	0.502 ^f^
CRP, mg/dL ^a,b^	3 (1.65, 5.2)	3 (2, 6)	2 (1, 3)	0.026 ^e^
P, mg/dL ^a,b^	3.6 (3.19, 4.35)	3.7 (3.11, 4.93)	3.55 (3.28, 3.86)	0.741 ^e^
PTH, pg/mL ^a,b^	55.25 (37.9,105.4)	77.2 (46.7,153.3)	41.85 (33.6, 50.8)	<0.001 ^e^
Ca × P ratio ^a,b^	32.81 (28.67, 41.61)	32.90 (28.83, 43.91)	32.34 (28.7, 39.18)	0.772 ^e^
NT-proBNP, pg/mL ^a,b^	191.3 (95.1, 379.45)	225.5 (99.1, 447.8)	139.65 (58.2, 205.95)	0.007 ^e^
Uric acid, mg/dL ^a,b^	6.19 (5.05, 7.2)	6.43 (5.77, 7.27)	4.72 (3.64, 6.66)	0.006 ^e^
Urea protein to creatinine ratio, mg/dL ^a,b^	0.12 (0.06, 0.28)	0.21 (0.08, 0.66)	0.07 (0.052, 0.17)	0.002 ^e^
Hb, g/dL ^c,d^	13.08 ± 1.70	12.70 ± 1.77	13.88 ± 1.24	0.007 ^g^
Albumin, g/L ^c,d^	3.82 ± 0.44	3.78 ± 0.46	3.92 ± 0.40	0.237 ^g^
Total cholesterol, mg/DL ^c,d^	215.21 ± 48.76	213.52 ± 54.75	218.59 ± 34.67	0.694 ^g^
HDL-cholesterol, mg/dL ^c,d^	44.6 ± 10.29	43.76 ± 9.89	46.32 ± 11.12	0.354 ^g^
LDL-cholesterol, mg/dL ^c,d^	97.73 ± 25.66	96.38 ± 26.79	100.18 ± 23.86	0.580 ^g^
Ca, mg/dL ^c,d^	9.19 ± 0.92	9.19 ± 1.01	9.18 ± 0.74	0.994 ^e^

Comments: CKD+—patients with eGFR ≤ 60 mL/min/1.73 m^2^; CKD–—patients with eGFR > 60 mL/min/1.73 m^2^; eGFR—estimated glomerular filtration rate; Hb—haemoglobin; CRP—C reactive protein; Ca—serum level of calcium; P—serum level of phosphorus; PTH—parathyroid hormone; NT-proBNP—N-terminal pro b-type natriuretic peptide; ^a^ Variables of non-normal distribution; ^b^ numbers are given as median (lower quartile, upper quartile); ^c^ variables of normal distribution; ^d^ numbers are given as mean ± SD; ^e^ the Mann–Whitney *U* test; ^f^ log transformation, Student’s *t*-test; ^g^ Student’s *t*-test.

**Table 3:** Echocardiographic parameters in both groups.

Parameter	Total *n* = 67	CKD+ *n* = 45	CKD– *n* = 22	*p* ^e^
IVSd ^a,b^	1.1 (1, 1.3)	1.1 (1, 1.3)	1 (0.9, 1.2)	0.032
LVIDd ^a,b^	4.6 (4.3, 4.85)	4.65 (4.4, 4.8)	4.4 (4.2, 5.1)	0.734
LVPWd ^a,b^	1.1 (1, 1.2)	1.1 (1, 1.2)	1 (0.9, 1.2)	0.152
%FS ^a,b^	30 (27, 34.5)	30 (27, 33)	31 (27, 37)	0.302
LVs Mass IND(ASE) ^a,b^	92.64 (80.6, 108.51)	97.2 (83, 111.1)	86.33 (75, 103.56)	0.160
Ao Diam ^a,b^	3.4 (3.2, 3.6)	3.4 (3.2, 3.5)	3.4 (3.3, 3.6)	0.828
MV E Vel ^a,b^	0.63 (0.55, 0.74)	0.6 (0.51, 0.71)	0.68 (0.63, 0.76)	0.031
MV E/A Ratio ^a,b^	0.83 (0.67, 1.02)	0.71 (0.63, 0.95)	0.96 (0.82, 1.2)	0.005
EF(Teich) ^c,d^	58.35 ± 6.92	57.64 ± 6.09	59.76 ± 8.32	0.300
LA Diam ^c,d^	4.0 ± 0.52	4.06 ± 0.54	3.88 ± 0.47	0.203
RVIDd ^c,d^	2.68 ± 0.29	2.7 ± 0.26	2.64 ± 0.34	0.464
MV A Vel ^c,d^	0.78 ± 0.17	0.8 ± 0.17	0.73 ± 0.17	0.064

Comments: CKD+—patients with eGFR ≤ 60 mL/min/1.73 m^2^; CKD–—patients with eGFR > 60 mL/min/1.73 m^2^; IVSd—interventricular septal diastolic diameter; LVIDd—left ventricular internal diameter end diastole; LVPWd—left ventricular posterior wall dimension at diastole; LVEF—left ventricular ejection fraction; LVFS—left ventricular fractional shortening; LVMI—left ventricular mass index; Ao Diam—aortic root diameter; LA Diam—left atrium diameter; RVIDd—right ventricular internal dimension in diastole; MV E Vel—mitral valve diastolic filling velocity; MV A Vel—mitral valve late diastolic filling velocity; ^a^ Variables of non-normal distribution; ^b^ numbers are given as median (lower quartile, upper quartile); ^c^ variables of normal distribution; ^d^ numbers are given as mean ± SD; ^e^ the Mann–Whitney *U* test.

**Table 4:** Comparison of pharmacological therapy in both groups.

Drug	Total *n* = 64	eGFR 0–29 *n* = 17		eGFR 30–60 *n* = 27		eGFR > 60 *n* = 20		*p* ^a^
		OH+ *n* = 6	OH– *n* = 11	OH+ *n* = 10	OH– *n* = 17	OH+ *n* = 7	OH– *n* = 13	
β-blockers, *n* (%)	24 (35.82)	4 (66.67)	4 (36.36)	7 (70.00)	3 (17.65)	3 (42.86)	3 (23.08)	0.040
ACE-I, *n* (%)	34 (50.75)	4 (66.67)	4 (36.36)	8 (80.00)	10 (58.82)	4 (57.14)	4 (30.77)	0.246
AT1-blockers, *n* (%)	7 (10.45)	1 (16.67)	1 (9.09)	1 (10.00)	2 (11.76)	0 (0.00)	2 (15.38)	0.100
Diuretics, *n* (%)	40 (59.70)	5 (83.33)	9 (81.82)	8 (80.00)	13 (76.47)	2 (28.57)	3 (23.08)	0.550
Ca-blockers, *n* (%)	25 (37.31)	4 (66.67)	6 (54.55)	4 (40.00)	7 (41.18)	0 (0.00)	4 (30.77)	0.171
Statins, *n* (%)	26 (38.81)	2 (33.33)	5 (45.45)	6 (60.00)	8 (47.06)	2 (28.57)	3 (23.08)	0.630

Comments: OH+—patients with orthostatic hypotension; OH–—patients without orthostatic hypotension; eGFR—estimated glomerular filtration rate; ACE-I—angiotensin converting enzyme inhibitors; AT1-blockers—angiotensin II receptor antagonists; Ca-blockers—calcium channel blockers; ^a^ Chi-squared MH (Mantel-Haenszel) test.

**Table 5:** Results obtained with the use of association rules in patients with eGFR ≤ 60 mL/min/1.73 m^2^.

Drug	Total *n* = 44	OH+ *n* = 16	OH– *n* = 28	*p* ^a^
β-blockers, *n* (%)	18 (49.91)	11 (68.75)	7 (25.00)	0.005
β-blockers +AH, *n* (%)	17 (38.64)	11 (68.75)	6 (21.43)	0.002
β-blockers +AH + diuretics, *n* (%)	16 (36.36)	10 (62.50)	6 (21.43)	0.007

Comments: AH—arterial hypertension; eGFR—estimated glomerular filtration rate; OH+—patients with orthostatic hypotension; OH–—patients without orthostatic hypotension; ^a^ Cramér’s V test.

**Table 6:** Results obtained with the use of association rules in patients with eGFR ≤ 60 mL/min/1.73 m^2^ and eGFR > 60 mL/min/1.73 m^2^.

Drug	Total *n* = 64	OH+ Group *n* = 23	OH– Group *n* = 41	*p*
β-blockers + CHF, *n* (%)	15 (23.44)	10 (43.48)	5 (12.20)	0.012 ^a^
β-blockers + CHF + eGFR ≤ 60 mL/min/1.73 m^2^, *n* (%)	10 (15.63)	8 (34.78)	2 (4.88)	0.005 ^a^
β-blockers + CHF + AH, *n* (%)	15 (23.44)	10 (43.48)	5 (12.20)	0.012 ^a^
β-blockers + CHF + comorbidity >1, *n* (%)	15 (23.44)	10 (43.48)	5 (12.20)	0.012 ^a^
β-blockers + CHF + diuretics, *n* (%)	12 (18.75)	8 (34.78)	4 (9.76)	0.033 ^a^
β-blockers + eGFR ≤ 60 mL/min/1.73 m^2^ + ACE-I, *n* (%)	12 (18.75)	8 (34.78)	4 (9.76)	0.033 ^a^
β-blockers + AH, *n* (%)	23 (35.94)	14 (60.87)	9 (21.95)	0.002 ^b^

Comments: CHF—congestive heart failure; AH—arterial hypertension; OH+—patients with orthostatic hypotension; OH–—patients without orthostatic hypotension; eGFR—estimated glomerular filtration rate; ^a^ Yates’s chi-squared test; ^b^ Cramér’s V test; comorbidity according to the Davies score [18].

**Table 7:** Results obtained with the use of association rules in male patients with eGFR ≤ 60 mL/min/1.73 m^2^ and eGFR > 60 mL/min/1.73 m^2^.

Drug	Total *n* = 30	OH+ Group *n* = 8	OH– Group *n* = 22	*p* ^a^
Comorbidity > 1 + ACE-I + eGFR ≤ 60 mL/min/1.73 m^2^, *n* (%)	10 (33.33)	6 (75.00)	4 (18.18)	0.013
ACE-I + eGFR ≤ 60 mL/min/1.73 m^2^, *n* (%)	14 (46.67)	7 (87.50)	7 (31.82)	0.022

Comments: OH+—patients with orthostatic hypotension; OH–—patients without orthostatic hypotension; eGFR—estimated glomerular filtration rate; ACE-I—angiotensin converting enzyme inhibitors; ^a^ Yates’s chi-squared test; comorbidity according to the Davies score [18].

**Table 8:** Multivariate logistic regression analysis in the group with eGFR ≤ 60 mL/min/1.73 m^2^.

Parameter	Odds Ratio	95% CI	*p*
CHF	15.31	1.44–162.44	0.020
β-blocker therapy	13.86	1.54–124.4	0.015
BP–standing position	0.91	0.84–0.97	0.003

Comments: CHF—congestive heart failure; BP—arterial blood pressure values; CI—confidence interval.
